# Protective Effects of H_2_S Donor Treatment in Experimental Colitis: A Focus on Antioxidants

**DOI:** 10.3390/antiox12051025

**Published:** 2023-04-28

**Authors:** Szilvia Török, Nikoletta Almási, Médea Veszelka, Denise Börzsei, Renáta Szabó, Csaba Varga

**Affiliations:** Department of Physiology, Anatomy and Neuroscience, Faculty of Science and Informatics, University of Szeged, H-6726 Szeged, Hungary

**Keywords:** hydrogen sulfide, inflammation, inflammatory bowel disease, colitis, peroxiredoxins, antioxidants

## Abstract

Inflammatory bowel diseases (IBD) are chronic, inflammatory disorders of the gastrointestinal (GI) system, which have become a global disease over the past few decades. It has become increasingly clear that oxidative stress plays a role in the pathogenesis of IBD. Even though several effective therapies exist against IBD, these might have serious side effects. It has been proposed that hydrogen sulfide (H_2_S), as a novel gasotransmitter, has several physiological and pathological effects on the body. Our present study aimed to investigate the effects of H_2_S administration on antioxidant molecules in experimental rat colitis. As a model of IBD, 2,4,6-trinitrobenzenesulfonic acid (TNBS) was used intracolonically (i.c.) to induce colitis in male Wistar–Hannover rats. Animals were orally treated (2 times/day) with H_2_S donor Lawesson’s reagent (LR). Our results showed that H_2_S administration significantly decreased the severity of inflammation in the colons. Furthermore, LR significantly suppressed the level of oxidative stress marker 3-nitrotyrosine (3-NT) and caused a significant elevation in the levels of antioxidant GSH, Prdx1, Prdx6, and the activity of SOD compared to TNBS. In conclusion, our results suggest that these antioxidants may offer potential therapeutic targets and H_2_S treatment through the activation of antioxidant defense mechanisms and may provide a promising strategy against IBD.

## 1. Introduction

Inflammatory bowel diseases (IBD) are chronic, inflammatory disorders of the gastrointestinal (GI) system which have become a global disease over the past few decades [1]. IBD comprises two main types: Crohn’s disease (CD) and ulcerative colitis (UC). CD and UC are intermittent diseases with the cyclic alternation of relapse and remission states and are characterized by many similar symptoms. However, the development and manifestation of these diseases are different [2]. CD can affect any part of the GI tract with transmural inflammation [3], while UC shows continuous superficial damage, which localizes in the colon [4]. Although currently used medications are commonly effective, they often cause side effects, and the long-term usage of these drugs is not recommended [5]. Therefore, new therapeutic targets and the development of new treatments are needed.

Even though IBD has been intensively researched, its pathogenesis is not fully understood. However, it is increasingly clear that oxidative stress is involved in the development and progression of IBD [6]. Under oxidative stress conditions, the oxidant/antioxidant homeostasis is disturbed through an excessive release of reactive oxygen species (ROS). During cellular oxygen metabolism, ROS are continuously produced as a byproduct in the body, including free radicals such as the superoxide anion (O_2_^•−^), hydroxyl radical (OH^•−^), non-radical hydrogen peroxide (H_2_O_2_) and hypochlorous acid (HOCl). Furthermore, ROS in analog with reactive nitrogen species (RNS) such as nitric oxide (NO), nitrogen dioxide (NO_2_), and peroxynitrite (ONOO^−^) are derived from nitrogen [7]. Nitrotyrosine (3-NT), which is formed through post-translational protein modification on free tyrosine residues by ONOO^−^, can be considered a marker of oxidative or nitrosative stress [8]. At an optimal concentration, ROS act as regulators of several signaling pathways; however, the accumulation of ROS in cells can cause reversible or irreversible structural and functional damage in many cell components and macromolecules, especially membrane lipids, proteins, and DNA [7]. Consequently, increased and uncontrolled ROS production leads to the injury of the mucosal barrier and the invasion of opportunistic pathogens contributing to inflammatory processes and tissue destruction in IBD [9]. The maintenance of intracellular redox homeostasis is mainly regulated by an endogenous antioxidant defense system, which consists of non-enzymatic and enzymatic antioxidants [7]. The major intracellular non-enzymatic compound is the glutathione (GSH), which can directly scavenge ROS, including O_2_^•−^, OH^•−^, and ONOO^−^, which serves as a co-factor for glutathione peroxidases (GPXs) in the detoxification processes of H_2_O_2_, and lipid hydroperoxides [10]. Furthermore, the most important defense mechanisms to avoid ROS-mediated damages are controlled by antioxidant enzymes, such as superoxide dismutases (SODs), GPXs, catalases (CATs), and peroxiredoxins (Prdxs) [11]. SODs play a central role in the protection against oxidative stress. These enzymes catalyze the dismutation of O_2_^•−^ by converting it to H_2_O_2_, and furthermore, by removing O_2_^•−^, the enzyme inhibits the generation of ONOO^−^ from O_2_^•−^ and NO [12]. CATs and GPXs can transform H_2_O_2_ into water [13]. Additionally, Prdxs, a ubiquitous family of thiol-dependent peroxidases, neutralize H_2_O_2_, hydroperoxides, and ONOO^−^, protecting cells from oxidative damage. In mammalian cells, six Prdx isoforms have been identified (Prdx1–6) [14]. From these, the roles of Prdx1 [15], -2 [16], -4 [17], and 6 [18] have been suggested to be implicated in IBD. Several studies have demonstrated, in line with the excessive generation of reactive free radicals in IBD, how the levels of endogenous antioxidants and antioxidant enzymes in colonic tissues are decreased [19]. Therefore, the enhancement of antioxidant defense may represent an effective therapy for IBD.

Based on the research of the last two decades, H_2_S is now accepted as an important signaling gas molecule in the body, along with nitric oxide (NO) and carbon monoxide (CO) [20]. Endogenous H_2_S is synthesized both enzymatically and non-enzymatically, and primarily during the metabolism of cysteine via transsulfuration pathways [21]. H_2_S has several molecular targets, such as different ion channels, receptors, proteins, and enzymes, by which the gasotransmitter can regulate a wide range of physiological and pathological processes [22]. It is increasingly clear that H_2_S plays a role in maintaining redox status and promoting defense mechanisms through the upregulation of antioxidant components or directly scavenging ROS [23]. Even though the antioxidant function of H_2_S has been extensively investigated, its effects on the isoforms of the Prdx family are still unclear.

Therefore, in the present study, we hypothesized that the protective effect of H_2_S may rely on the activation of endogenous antioxidant signaling pathways in 2,4,6-trinitrobenzenesulfonic acid (TNBS)-induced rat colitis. Primarily we focused on the alteration of antioxidant GSH, SOD, and four isoforms of Prdxs due to an exogenously administered H_2_S donor. To the best of our knowledge, this is the first study to investigate the relationship between H_2_S and Prdxs in IBD. Our results reveal that H_2_S donor treatment can significantly suppress the levels of the oxidative stress marker 3-NT while significantly elevating levels of GSH, Prdx1, Prdx6, and SOD activity in the inflamed colon. Based on our results, we suggested H_2_S administration as a promising strategy for IBD treatment by protecting against oxidative stress.

## 2. Materials and Methods

### 2.1. Experimental Animals

All experimental procedures were performed under the recommendations of the European Community guidelines for the Care and Use of Laboratory Animals and were approved by the Institutional Ethics Committee (XX./4799/2015, 15 December 2015) at the University of Szeged.

Male Wistar–Hannover rats (225–250 g) were purchased from Toxi-Coop Ltd. (Budapest, Hungary) and were kept under controlled circumstances with a 12 h light/dark cycle. Animals were provided with standard rat chow and tap water ad libitum.

### 2.2. Preparation of Drugs

Chemicals were purchased from Sigma-Aldrich (St. Louis, MO, USA) and prepared directly before use. For the induction of colitis, 2,4,6-trinitrobenzenesulfonic acid (TNBS) was dissolved in 50% ethanol (EtOH). As an H_2_S donor, Lawesson’s reagent (LR) was suspended in 0.5% carboxymethylcellulose (CMC). Animals were anesthetized with thiopental (Tiobarbital Braun, 0.5 g, B. Braun Medical SA, Barcelona, Spain) and dissolved in physiological saline (0.9%).

### 2.3. Induction of Colitis and Experimental Design

The animals were assigned to three groups: an absolute control (Ctrl, no treatment, *n* = 8), EtOH group (0.25 mL 50% ethanol, *n* = 8), and TNBS group (10 mg in 0.25 mL of 50% ethanol, *n* = 30). The induction of colitis was based on the method described by Morris et al. [24]. Briefly, after overnight fasting, a single dose of TNBS was administered to the animals intracolonically (i.c.) with an 8 cm long polyethylene cannula under mild anesthesia (thiopental, i.p. 40 mg/kg). TNBS-treated animals were separated into different groups and treated per os twice a day with the following drugs: Lawesson’s reagent (LR, an H_2_S donor, *n* = 12) at the dose of 18.75 µM/kg/day; carboxymethylcellulose (CMC, 0.5%, vehicle of Lawesson’s reagent, *n* = 8). The treatment doses were based on our prior results in the same colitis model [25]. After 72 h of TNBS instillation, the animals were euthanized (thiopental, i.p. 100 mg/kg), and a distal 8 cm section of the colon was harvested, opened, rinsed immediately in cold physiological saline, and pictures were taken of it (Panasonic Lumix DMC-TZ6, Panasonic Co., Kadoma, Osaka, Japan) to determine macroscopic inflammatory damage. Finally, the colon tissues were frozen, powdered in a mortar with a pestle in liquid nitrogen, and kept at −80 °C until biochemical measurements.

### 2.4. Determination of Macroscopic Damage

The severity of inflammation and ulceration was determined macroscopically in a randomized manner from the colon photos, using a proprietary computerized planimetry software that was evolved in our laboratory (Stat_2_1_1, Szeged, Hungary). The extent of the macroscopically apparent mucosal injury was calculated and presented as the percentage (%) of the total 8 cm tissue segment area.

### 2.5. Measurement of SOD Activity in the Colon Tissue

The colonic SOD activity was determined with a kit from Abcam (Cambridge, UK). The samples were homogenized (Benchmark Scientific Handheld homogenizer D1000, Benchmark Scientific, New Jersey, MA, USA) in ice-cold 0.1 M Tris/HCL, pH 7.4 containing 0.5% Triton X-100, 5 mM β-ME, and 0.1 mg/mL PMSF, which were then centrifuged at 14,000× *g* for 5 min at +4 °C. Supernatants were collected, and the assay was performed according to the instructions of the manufacturer. Optical densities (OD) were measured at 450 nm on a microplate reader. Finally, the SOD activity (inhibition rate %) was calculated using the following equation:

(1) $$\mathrm{SOD}\mathrm{Activity}(\mathrm{inhibition}\mathrm{rate}\%)=\frac{(\mathrm{Ablank1}-\mathrm{Ablank3})-(\mathrm{Asample}-\mathrm{Ablank2})\times 100}{\left(\mathrm{Ablank1}-\mathrm{Ablank3}\right)}\;A=\mathrm{absorbance}$$

### 2.6. Measurement of Colonic Total GSH Level

To measure the colonic total GSH (GSH + GSSG) levels, tissues were homogenized in a solution containing 0.25 M sucrose, 1 mM DTT, and 20 mM Tris and then centrifuged at 15,000× *g* for 30 min at +4 °C. Supernatants were incubated in a solution of 0.1 M CaCl_2_, 0.25 M sucrose, 20 mM Tris, and 1 mM DTT for 30 min at 0 °C. Then, the samples were further centrifuged at 21,450× *g* for 60 min at +4 °C, and the clear cytosolic fractions were used for the enzyme assay. A mixture of 125 mM Na phosphate and 6.0 mM EDTA was used for the stock solution of GSH, glutathione reductase, 5,5′dithio-bis-2-nitrobenzoic acid (DTNB), and β-NADPH. In a 96-well microplate, 40 μL of each blank, standard, or sample in the form of 20 μL of a DTNB stock solution and 140 μL of β-NADPH were added to each well and incubated for 5 min at +25 °C. To start the reaction, a 10 μL volume of glutathione reductase was used, and after 10 min of shaking, the absorbance was measured at 405 nm. In the assay, GSH was sequentially oxidized with DTNB and reduced by NADPH in the presence of glutathione reductase. The total GSH levels were expressed in nmol/mg protein.

### 2.7. Measurement of Colonic 3-NT and Prdxs Levels by ELISA

Double antibody sandwich ELISA kits were used for the determination of tissue levels of 3-NT (Bioassay Technology Laboratory, Shanghai, China) and four isoforms of Prdxs (Prdx1, -2, -4, -6) (GenAsia Biotech Co., Ltd., Shanghai, China). The colon samples were homogenized (Benchmark Scientific Handheld homogenizer D1000, Benchmark Scientific, New Jersey, MA, USA) in an ice-cold Phosphate Buffer Saline (PBS, pH 7.4) and centrifuged at 3000 rpm for 20 min at +4 °C. The measurements were carried out according to the manufacturer’s instructions and protocols, and ODs were determined spectrophotometrically at 450 nm. The results are expressed in nmol/L (3-NT) or pg/g protein (Prdx1, -2, -4, -6).

### 2.8. Determination of Protein Concentrations

The total protein contents were measured by the Bradford assay. To perform the measurement, 200 μL of the Bradford reagent was added to the bovine serum albumin (BSA) standard dilutions and each appropriately diluted sample. The protein concentration of the samples was assayed spectrophotometrically at 595 nm and expressed as mg protein/mL.

### 2.9. Statistical Analysis

All values were represented as the mean ± standard error of the mean (S.E.M.). Statistical analysis was performed by SigmaPlot 12.0 for Windows (Systat Software Inc., San Jose, CA, USA). Normality was checked in all datasets with a Shapiro–Wilk test. Parametric data were assessed by one-way ANOVA followed by a Holm–Sidak post hoc test, and these were nonparametric with the Kruskal–Wallis test followed by Dunn’s post hoc test. For all analyses, a probability (*p*) < 0.05 was accepted as a statistically significant difference.

## 3. Results

### 3.1. Hydrogen Sulfide Donor Ameliorates the Extent of Lesions in TNBS-Induced Rat Colitis

**Figure 1:**
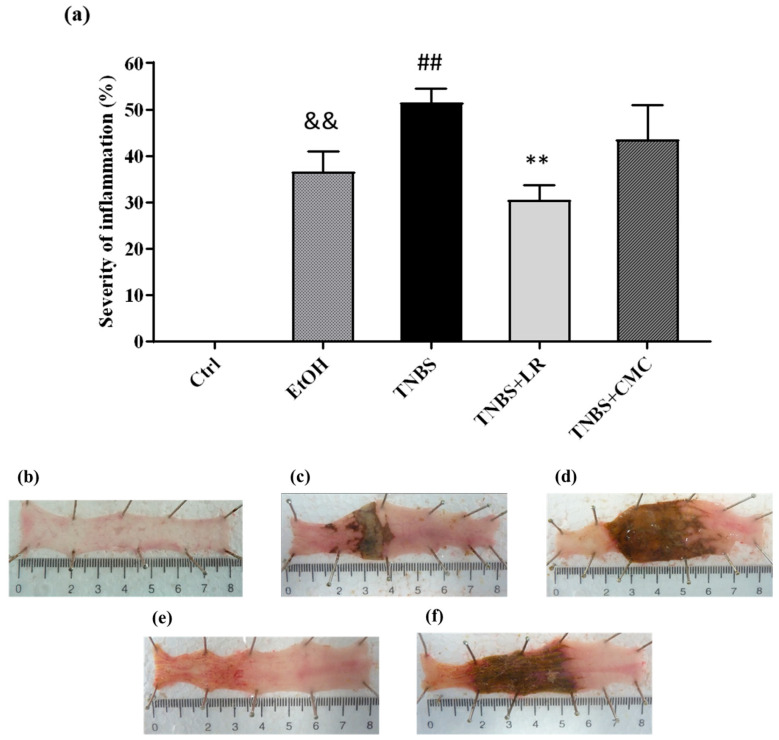
(**a**) Visible colonic lesions in the different groups of TNBS-induced rat colitis. Representative images of colonic inflammation in the experimental groups: (**b**) Ctrl (absolute control, no treatment); (**c**) EtOH (50% ethanol, the solvent of TNBS); (**d**) TNBS (2,4,6-trinitrobenzenesulfonic acid enema); (**e**) TNBS + LR (TNBS + 18.75 µM/kg/day Lawesson’s reagent); (**f**) TNBS + CMC (TNBS + 0.5% carboxymethylcellulose, the vehicle of Lawesson’s reagent). Results are shown as mean ± S.E.M.; *n* = 6–12/group; one-way ANOVA, Holm–Sidak post hoc test, and && *p* < 0.001 Ctrl vs. EtOH; ## *p* < 0.001 Ctrl vs. TNBS; ** *p* < 0.001 TNBS vs. TNBS + LR.

To model colitis, the animals were injected intracolonically with 2,4,6-trinitrobenzenesulfonic acid (TNBS) dissolved in 50% ethanol (EtOH), and 72 h after the administration, macroscopic colonic damages were analyzed. Compared to the untreated healthy controls, both TNBS and its solvent EtOH caused serious mucosal lesions in the colon tissues. As the dissolvent of TNBS, EtOH alone induced hyperaemia and superficial ulcers; however, the administration of TNBS resulted in more pronounced colonic injuries with severe hemorrhagic necrosis. The extent of the inflammation was approximately 50% of the examined colonic area in the TNBS group (51.58 ± 2.97 %). Treatment with an effective dose of H_2_S donor Lawesson’s reagent (LR, 18.75 µM/kg/day) significantly attenuated the severity of colon lesions compared to the tissues from the TNBS group (30.60 ± 3.08 vs. 51.58 ± 2.97%). The vehicle of LR, CMC, was also examined alone, and there was no statistically significant difference in mucosal injury compared to the TNBS group, suggesting that the anti-inflammatory effect was a consequence of H_2_S released from LR (Figure 1).

### 3.2. Hydrogen Sulfide Donor Attenuated the Level of Oxidative Stress Marker 3-NT

**Figure 2:**
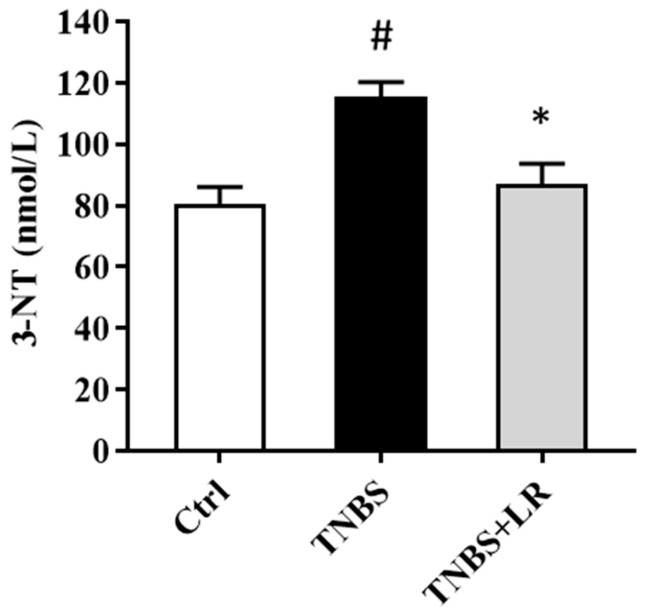
Effect of LR on the level of oxidative stress marker 3-NT in rat colitis. Ctrl (absolute control, no treatment); TNBS (2,4,6-trinitrobenzenesulfonic acid enema); TNBS + LR (TNBS + 18.75 µM/kg/day Lawesson’s reagent). Results are shown as mean ± S.E.M.; *n* = 8/group; one-way ANOVA, Holm–Sidak post hoc test, # *p* < 0.05 Ctrl vs. TNBS; * *p* < 0.05 TNBS vs. TNBS + LR.

As a marker of oxidative stress, the level of 3-NT was significantly elevated after the induction of colitis by TNBS compared to the control group (115.58 ± 4.63 vs. 80.34 ± 5.76 nmol/L). The administration of an effective dose of the H_2_S donor significantly attenuated the level of the stress marker compared to the TNBS group (86.83 ± 6.81 vs. 115.58 ± 4.63 nmol/L). The results are presented in Figure 2.

### 3.3. Effect of Hydrogen Sulfide Donor on the Level of Antioxidant Total GSH in TNBS Colitis

**Figure 3:**
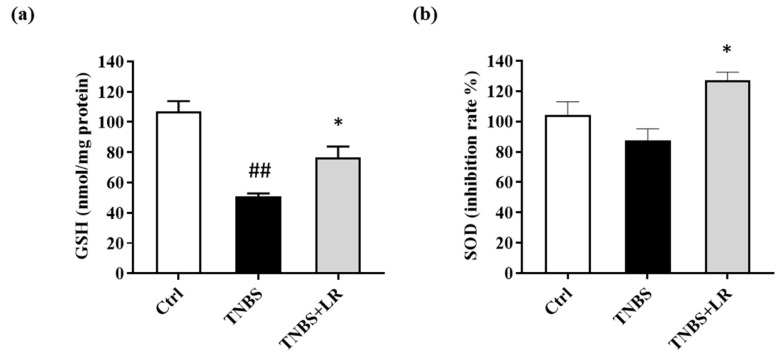
Changes in the (**a**) total level of antioxidant GSH and (**b**) SOD activity after LR treatment in TNBS-induced colitis. Ctrl (absolute control, no treatment); TNBS (2,4,6-trinitrobenzenesulfonic acid enema); TNBS + LR (TNBS + 18.75 µM/kg/day Lawesson’s reagent). Results are shown as mean ± S.E.M.; *n* = 5–12/group; one-way ANOVA, Holm–Sidak post hoc test, ## *p* < 0.001 Ctrl vs. TNBS; * *p* < 0.05 TNBS vs. TNBS + LR.

Concerning the antioxidant defense mechanisms, the total GSH level was measured. Following TNBS administration, the GSH content was significantly diminished compared to the untreated control animals (50.73 ± 2.06 vs. 106.86 ± 6.94 nmol/mg protein). As a result of LR treatment, the level of GSH was increased in comparison with the TNBS group, and this difference was statistically significant (76.69 ± 7.16 vs. 50.73 ± 2.06 nmol/mg protein) (Figure 3a).

### 3.4. Hydrogen Sulfide Donor Treatment Elevated the SOD Activity in TNBS-Induced Colitis

As shown in Figure 3b, a slight reduction was observed in the activity of SOD after the induction of colitis (87.68 ± 7.51 vs. 104.19 ± 8.76 inhibition rate%), but there was no significant difference between TNBS and the untreated control groups. However, the release of H_2_S from LR caused a significant elevation in the activity of the antioxidant enzyme compared to the TNBS group (127.12 ± 5.36 vs. 87.68 ± 7.51 inhibition rate %).

### 3.5. Alterations in the Levels of Prdx1, -2, -4, and -6 after Hydrogen Sulfide Donor Treatment in TNBS Colitis

**Figure 4:**
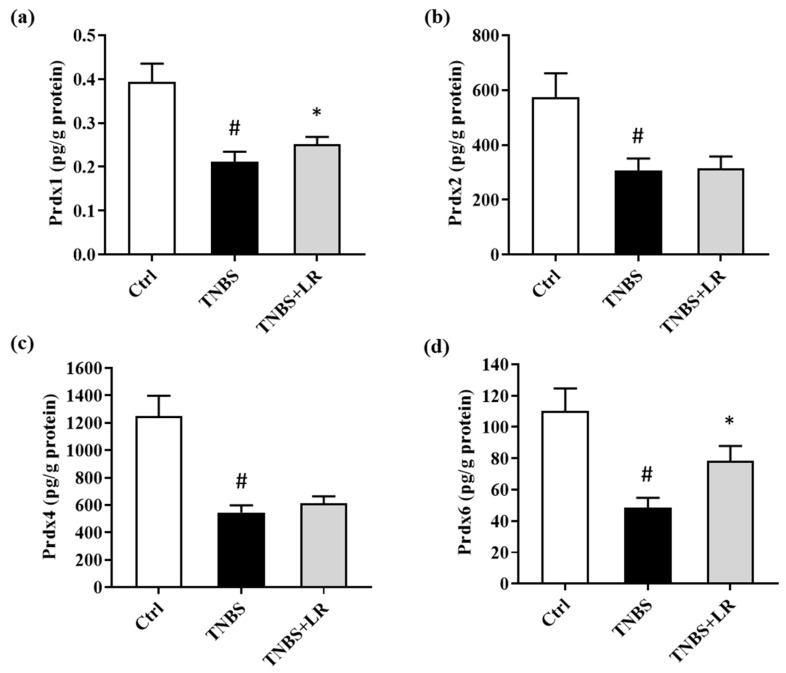
Alterations in the level of (**a**) Prdx1, (**b**) Prdx2, (**c**) Prdx4, (**d**) Prdx6 due to LR treatment in TNBS colitis. Ctrl (absolute control, no treatment); TNBS (2,4,6-trinitrobenzenesulfonic acid enema); LR (TNBS + 18.75 µM/kg/day Lawesson’s reagent). Results are shown as mean ± S.E.M.; *n* = 7–11/group; one-way ANOVA, Holm–Sidak post hoc test or Kruskal–Wallis test followed by Dunn’s post hoc test, # *p* < 0.05 Ctrl vs. TNBS; * *p* < 0.001 TNBS vs. TNBS + LR.

In the present study, we investigated alterations in the levels of Prdx1, -2, -4, and -6 isoforms of Prdx antioxidant enzymes after the induction of colitis and H_2_S donor treatment. TNBS administration significantly reduced the levels of all measured isoforms compared to the absolute control group (0.18 ± 0.01 vs. 0.39 ± 0.04; 306.58 ± 44.05 vs. 574.17 ± 87.52; 546,40 ± 51.80 vs. 1250.46 ± 146.96; 48.62 ± 6.17 vs. 110.16 ± 14.48 pg/g protein). However, in the case of Prdx1 and Prdx6, we observed a statistically significant elevation as a result of LR treatment compared to the TNBS group (0.25 ± 0.02 vs. 0.18 ± 0.01; 78.36 ± 9.60 vs. 48.62 ± 6.17 pg/g protein). Our findings showed that H_2_S did not affect the levels of Prdx2 and Prdx4 isoforms. The results are presented in Figure 4.

## 4. Discussion

Oxidative stress-mediated signaling mechanisms were implicated in the pathogenesis of several inflammatory diseases, such as IBD [7,26]. In the present study, we investigated the effects of H_2_S donor Lawesson’s reagent (LR) treatment on the components of the endogenous antioxidant defense system in a chemical-induced model of IBD. Our results show that the oral administration of H_2_S ameliorated the severity of inflammation in TNBS-induced rat colitis. Additionally, the H_2_S donor significantly reduced the tissue level of oxidative stress marker 3-NT, and in the case of antioxidants, it resulted in a significant increase in the level of GSH and the activity of SOD. Moreover, the levels of Prdx1 and Prdx6 antioxidant enzymes were also significantly elevated after H_2_S donor treatment. Our findings suggest that the protective effect of H_2_S may partially rely on preventing the harmful effects of oxidative stress through the induction of various antioxidants.

The number of patients with IBD is continuously increasing worldwide, but its therapy has still not been completely resolved. Though the currently used conventional therapeutic options to reduce inflammation effectively, the long-term usage of these drugs is not recommended because of their common side effects [5]. Today, the participation of H_2_S as an important gasotransmitter in several physiological and pathological processes has been well established [27]. An increasing amount of evidence suggests that in physiological concentrations, H_2_S has a cytoprotective, antioxidant, vasodilator, anti-inflammatory, and anti-apoptotic effect [28]. Furthermore, several investigations reported the benefits of H_2_S donor molecules and their drug hybrids in a reduction in inflammation with fewer side effects, suggesting their therapeutic options in various diseases [29,30].

In our current study, a TNBS-induced colitis model was used and developed by Morris et al. [24], which is a widely accepted model for studying IBD. TNBS instillation induces Th1 and Th17 cell-mediated pathways and transmural inflammation with ulcers which are hallmarks of human CD [31]. In this study, we reported that H_2_S, when released from LR, significantly reduced the severity of inflammation and the area of ulceration in TNBS rat colitis. These results confirm our previous study in relation to the anti-inflammatory effect of H_2_S in the same colitis model [25]. Furthermore, our observations are consistent with the findings of Matsunami et al. [32]. They also found that the intracolonic administration of NaHS, an H_2_S donor, suppressed inflammation in the TNBS-induced rat colitis model. In dextran sulfate sodium (DSS)-induced mice colitis, Chen et al. [33] demonstrated that the intraperitoneal injection of NaHS decreased clinical symptoms, the level of pro-inflammatory parameters, as well as tissue damage. However, the background of the anti-inflammatory mechanisms of H_2_S is still not exactly clear.

The overproduction of ROS is crucial in inflammatory processes [26]. These reactive intermediates are produced in large amounts by infiltrating leucocytes, which contribute to tissue destruction [34]. In the present study, we found an elevation in the level of the oxidative stress marker 3-NT after the induction of colitis by TNBS. This result is in accordance with our previous observation in the same colitis rat model [35]. Furthermore, Gochman et al. [36] detected a higher expression of 3-NT in human biopsies of colitis and cancer compared to colonic sections from healthy patients. However, here we showed that repeated oral H_2_S donor administration resulted in a significant reduction in the level of this marker. Furthermore, Mishra et al. [37] found that NaHS treatment in mice with chronic heart failure attenuated 3-NT content in the heart tissue.

H_2_S can scavenge ROS directly; however, it can be presumed that the primary antioxidant mechanism of H_2_S is the promotion of intracellular antioxidant gene expression by sulfhydration key signaling molecules [38]. Previously, we showed that H_2_S donor treatment reduced inflammation through the activation of antioxidant and anti-inflammatory heme oxygenase 1 in experimental colitis [25]. One of the non-enzymatic antioxidants is GSH in the body, which has an important role in antioxidant defense [10]. In physiological circumstances, GSH occurs mainly in a reduced form, while under oxidative stress conditions, the oxidized form (GSSG) increases. Thus, the decreased GSH/GSSG ratio is a good indicator of oxidative stress [39]. Sido et al. reported decreased GSH and increased GSSG levels in the mucosa of patients suffering from IBD compared to the healthy control group [40]. In our study, we showed that the level of GSH decreased after the induction of colitis; however, the protective dose of the H_2_S donor significantly elevated the level of this antioxidant. Consistent with our current findings, Guo et al. [41] detected reduced GSH levels in ischemia–reperfusion (I/R)-induced gastric epithelial cells injury; however, H_2_S donor NaHS preconditioning on cells prevented I/R-related oxidative stress and inflammatory processes by increasing the GSH level and reducing ROS production. Furthermore, Cui et al. [42], in a gastric I/R rat model, found that pretreatment with an endogenous H_2_S synthetase blocker DL-propargylglycine (PAG) led to gastric mucosal ulceration in rats, but pretreatment with L-cysteine, the precursor of H_2_S, protected the mucosa from the damage induced by I/R. They measured decreased GSH levels in the gastric mucosal tissue from the PAG group, while L-cysteine increased the GSH content. Additionally, in rats pretreated with L-cysteine, increasing endogenous H_2_S resulted in the enhanced activity of the antioxidative enzyme, SOD, compared to decreased enzyme activity when the generation of H_2_S was blocked by PAG. Our results are in accordance with this observation since the exogenously administered H_2_S donor significantly elevated the activity of SOD, which was suppressed by TNBS in the rat colon. Seguí et al. [43] also demonstrated in the same colitis model that SOD treatment ameliorated the observed clinical, pathologic, and inflammatory markers of colitis and diminished lipid peroxidation in a dose-dependent manner in inflamed colonic tissues.

Prxs are highly conserved and abundant enzymes that are mainly involved in defense mechanisms against oxidative stress by eliminating peroxides [14]. However, the role of Prdxs in inflammatory disorders is controversial [16,44]. Here, we found that the levels of Prdx1, -2, -4, and -6 were significantly attenuated in TNBS-induced colitis. The current results confirm our previous observation in the same experimental model, where we also showed suppressed levels of the four measured Prdx isoforms [35]. Moreover, Hsieh et al. [45] demonstrated the downregulation of Prdx1 and Prdx2 in colonic biopsy samples from patients with UC. In contrast to our results, Wu et al. [46] showed an increased Prdx1 level in rats with dextran sulfate sodium (DSS)-induced colitis, and they found that the silencing of Prdx1 expression improved colonic damage induced by DSS. Moreover, Horie et al. [15] suggested Prdx1 as a potential marker for monitoring oxidative stress in UC. Additionally, Senhaji et al. [16] detected overexpression of Prdx2 in the samples of IBD patients. Furthermore, Tagaki et al. [17] reported higher disease activity index (DAI) scores and levels of inflammatory markers in Prdx4 knockout mice with colitis induced by DSS, suggesting the protective effect of Prdx4 in IBD. Melhem et al. [18] found in their study that Prdx6 was downregulated in acute and chronic DSS colitis in mice, which is in line with our findings in the case of Prdx6 in TNBS-induced rat colitis. However, they showed overexpression of Prdx6 mRNA in colonic samples of IBD patients. Interestingly, they also demonstrated that Prdx6 knockout mice with colitis had less ulceration and lower levels of pro-inflammatory parameters. In our present study, we observed changes in the four Prdx isoforms after H_2_S donor administration in TNBS-induced colitis. We found significant elevations in the enzyme contents in the case of Prdx1 and Prdx6 due to the H_2_S released from LR in the inflamed colons. However, H_2_S donor treatment did not affect the levels of Prdx2 and Prdx4. To the best of our knowledge, this is the first paper to describe the association between H_2_S treatment and Prdxs in IBD. In accordance with us, Liu et al. [47] showed that pretreatment with NaHS on H9c2 cardiomyocytes attenuated doxorubicin-induced cardiotoxicity and reversed increased expression levels of Prdx3. Additionally, Chen et al. [48] detected decreased Prdx2 levels in the D-gal-induced aging model which was reversed by NaHS. Furthermore, Unuma et al. [49] reported enhanced Prdx4 levels in an animal model of sepsis, but this increase was attenuated by the pretreatment of GYY4137, an H_2_S donor, which contributed to the protective effect of H_2_S.

### Limitation

Our results are promising; however, this study has some limitations. The first limitation is that we measured the total GSH level, although this was less informative about oxidative stress state than the GSH/GSSG ratio. Additionally, the second limitation of our research is that we did not investigate the comprehensive signaling pathways exerted by H_2_S supplementation. Therefore, it would be worth clarifying the exact signaling mechanism of the H_2_S-mediated antioxidant effect in the future.

## 5. Conclusions

In conclusion, we found that H_2_S donor treatment attenuated inflammation, which could contribute to ulcer healing in TNBS-induced rat colitis by activating antioxidant defense mechanisms. The GSH level, the activity of SOD, as well as Prdx1 and Prdx6 levels, were increased due to H_2_S administration, suggesting the protective actions of H_2_S against oxidative stress-induced tissue destruction. Based on our findings, these antioxidants may offer potential therapeutic targets, and H_2_S treatment may provide a new strategy against IBD. Our current result is promising; however, further investigations are needed to understand the precise role of Prdxs in inflammatory conditions and the protective mechanisms of H_2_S.

## Data Availability

All data analyzed in this study are included in this published article.
